# The Use of Polymer Inclusion Membranes for the Removal of Metal Ions from Aqueous Solutions—The Latest Achievements and Potential Industrial Applications: A Review

**DOI:** 10.3390/membranes12111135

**Published:** 2022-11-11

**Authors:** Małgorzata A. Kaczorowska

**Affiliations:** Faculty of Chemical Technology and Engineering, Bydgoszcz University of Science and Technology, 3 Seminaryjna Street, 85326 Bydgoszcz, Poland; malgorzata.kaczorowska@pbs.edu.pl

**Keywords:** polymer inclusion membrane, metal ions, polymer, plasticizer, carrier

## Abstract

The growing demand for environmentally friendly and economical methods of removing toxic metal ions from polluted waters and for the recovery of valuable noble metal ions from various types of waste, which are often treated as their secondary source, has resulted in increased interest in techniques based on the utilization of polymer inclusion membranes (PIMs). PIMs are characterized by many advantages (e.g., the possibility of simultaneous extraction and back extraction, excellent stability and high reusability), and can be adapted to the properties of the removed target analyte by appropriate selection of carriers, polymers and plasticizers used for their formulation. However, the selectivity and efficiency of the membrane process depends on many factors (e.g., membrane composition, nature of removed metal ions, composition of aqueous feed solution, etc.), and new membranes are systematically designed to improve these parameters. Numerous studies aimed at improving PIM technology may contribute to the wider use of these methods in the future on an industrial scale, e.g., in wastewater treatment. This review describes the latest achievements related to the removal of various metal ions by PIMs over the past 3 years, with particular emphasis on solutions with potential industrial application.

## 1. Introduction

Generation of large amounts of industrial wastewater, sludges and wastes containing hazardous heavy metals associated with the development of various industries (e.g., electroplating, oil and gas industry, steel industry, chemical industry, etc.) is a significant environmental issue [1,2,3]. Environmental contamination with heavy metals, which are toxic and non-biodegradable, poses a great risk, among others, to aquatic organisms, agricultural crops and humans, who can suffer serious health problems [4,5]. Limiting the emission of hazardous metals to the environment can be achieved by the introduction of regulations obliging manufacturers to treat wastewater/sludge/waste properly and develop safe and economical methods that can be used for this purpose [6]. As the impact of heavy metals on living organisms depends on many factors, including the type of metal and its concentration, acceptable limits (considered safe) were determined for the concentrations of individual heavy metals, e.g., in industrial wastewater, agricultural soils or in ground and drinking water. For particularly hazardous metals, such as arsenic, mercury or lead, the acceptable concentration limits, e.g., in drinking water, are very low [7,8,9], and this is why the methods of metal ion removal must be sufficiently effective. At the same time, they should be environmentally friendly [10,11]. Therefore, research is being carried out on the possibility of efficient removal of pollutants from water using new solutions, for example, novel, eco-friendly adsorbents, such as chitosan produced from shells of shrimp waste (imprinting and non-imprinting composites with Ulva lactuca algae intended for Cd(II) removal), granular activated carbon from biomass waste materials (removal of metal ions, pharmaceuticals, carcinogenic compounds, etc.) or reusable magnetic hydrophilic composites (metal organic framework/wood composites for synthetic dye removal) [12,13,14].

One of the groups of separation methods considered to be environmentally friendly and energy-saving are membrane techniques [15,16]. Technological advancements in the development of various types of membranes over the last decades have led to the development of materials that are not only characterized by high efficiency, but also selectivity in relation to specific metal ions, which is of great importance in the case of industrial applications [17]. A membrane, which is usually a thin layer, has been defined as a permeable or semi-permeable phase (solid, liquid, a solvent-swollen gel) that acts as a barrier between two adjacent phases (gas or liquid) and controls the exchange of substances between them [18,19]. In general, membrane processes can be classified in various ways, for example according to the types of membranes used (i.e., natural or synthetic, organic or inorganic, isotropic or anisotropic, etc.) or to the exerted driving force (i.e., equilibrium- or non-equilibrium-based membrane processes, pressure driven and non-pressure driven processes, etc.) [18,19]. Among the various types of membranes, liquid membranes are becoming more and more popular, especially polymer inclusion membranes (PIMs), in which the liquid phase is held within the polymeric network of a base polymer [20]. PIMs are a type of functionalized membrane that can be tailored to the properties of removed target analyte, characterized by many advantages such as the possibility of conducting simultaneous extraction and back extraction, excellent stability and high reusability, the simplicity and relatively low costs of membrane process, as well as low consumption of organic solvents (especially compared to classical extraction) [20,21]. Due to their versatility, they can be used for different purposes in analytical chemistry; however, they work especially well as an extraction medium for removing various substances, including pharmaceuticals, organic contaminants, as well as different anions and cations from aqueous solutions [20,22,23,24]. The results of research conducted in recent years clearly show that polymer inclusion membranes can be successfully used to remove hazardous metal ions from various types of wastewater and to recover noble metal ions from waste, e.g., from waste electrical and electronic equipment (WEEE) [25,26,27]. In the latter case, typically the WEEE is leached, and then PIMs are used to “transfer” the desired metal ions from leached liquor into a properly selected solution of much smaller volume. The extraction and stripping of the precious metal ions with the use of polymer inclusion membranes is relatively uncomplicated [27]. However, the use of PIMs to remove or recover specific metal ions from an aqueous solution allows obtaining satisfactory process efficiency and selectivity, but only with the proper selection of membrane components (and their amounts) and optimization of membrane process parameters (i.e., pH, temperature, types of receiving phases, etc.), which in many cases is not easy, both due to the properties of removed/recovered metal ions and the complexity of the industrial wastewater in which they occur [28,29,30]. 

**Figure 1:**
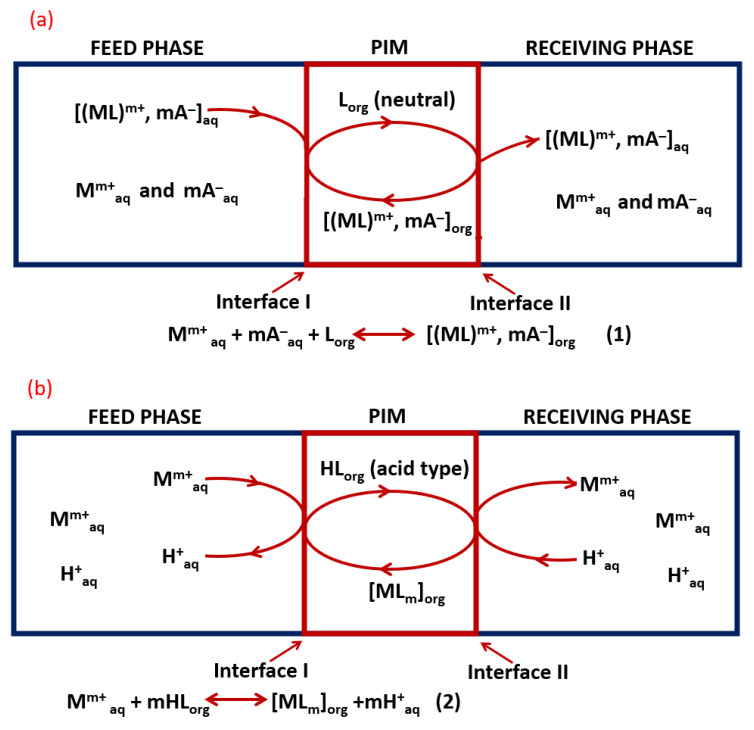
Scheme of transport of metal ions M^m+^ through PIMs: (**a**) facilitated co-transport (PIM with neutral carrier), (**b**) counter-facilitated transport (PIM with acid type carrier) (prepared on the basis of [33] and references therein).

The basic components of polymer inclusion membranes are a polymer that ensures the mechanical strength of the membrane and a carrier that is responsible for removing desired species, i.e., metal ions from the aqueous solution, and transporting them into the membrane structure. The structure and properties of the carrier are essential, as they influence the mechanism of the extraction process (chelation or ion exchange) and affect selective membrane permeability. The formed ion pair or the complex (analyte and carrier) can diffuse through the membrane, and then, using a properly selected receiving phase, metal ions are released and the carrier is able to repeat the transport process [20,24,31]. Depending on the type of carrier, transport of metal ions is subject to a facilitated co-transport (chelating-type carrier) and counter transport (acid-type carrier) [32]. Figure 1 shows simplified processes of facilitated co-transport and counter transport of metal ions M^m+^ through PIMs containing different types of carriers. In the case of co-transport (1a), associated ion pairs M^m+^ and mA^−^ (A—anions) contained in the feed phase are extracted, reversible by the neutral carrier L (component of the PIM). The processes taking place at the feed phase/membrane and membrane/receiving phase interfaces can be described by the Equation (1), and the metal cations and anions migrate in the same direction to the receiving phase. In the case of counter transport, when the carrier contained in the PIM is acid-type, the process is based on a cation exchange proton mechanism: cations migrate in the opposite direction to protons (from high pH to low pH solutions), and the equilibrium at the feed face/membrane interface can be described by Equation (2) ([33] and references therein).

**Figure 2:**
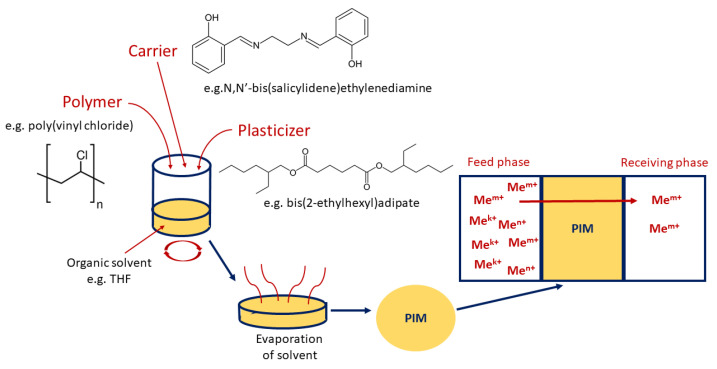
Scheme of formulation of polymer inclusion membrane by casting method (based on [35]).

In addition, during the formulation of PIMs, plasticizers which influence the physical and chemical properties of the membranes (i.e., improve their flexibility and affect the efficiency of the metal ions’ transport process) can be added. It has been shown that some carriers can also act as plasticizers [25]. Usually, PIMs are prepared by a solvent casting method, shown in Figure 2 (dissolving the membrane components in an organic solvent, e.g., THF, after thorough mixing, then pouring the solution on a self-levelling dish and evaporating the solvent); however, in the case of PIMs containing an ionic liquid as a carrier, it is possible to apply solvent-free procedures, for example based on a thermal compression technique [34].

Despite the systematically increasing applicability of PIMs in the removal/recovery of various metal ions, many similar metal ions are still difficult to separate with the use of polymer inclusion membranes [16]. Moreover, the successful utilization of certain PIMs in laboratory conditions does not guarantee their effectiveness and resistance to long-term use on an industrial scale. There are several different approaches to dealing with the problem of selectivity, stability and durability of PIMs. The most common ones are based on introduction of new extractants, polymers and plasticizers used during the formulation of membranes, but other solutions are also sought, e.g., manufacturing possibilities of multilayer polymeric membranes [32,35]. This article reviews the latest achievements (from 2020) in the use of PIMs for the removal of various metals from aqueous solutions and for the recovery of precious metals from leached waste, and shortly discusses the potential usefulness of these methods in industrial applications.

## 2. Traditional and Novel Polymeric Materials Used in Preparation of PIMs

Although various polymeric materials can be used for the formulation of PIMs, only some of them allow for obtaining a membrane with the desired properties, and due to their affordability/ease of synthesis and low costs, do not adversely affect the profitability of the membrane processes. The group of polymers widely used in the fabrication of PIMs includes, among others, polyvinyl chloride (PVC), cellulose triacetate (CTA), polysulfone (PSF) and polyvinylidene fluoride (PVDF). However, in order to improve the efficiency of PIMs, intensive research is also carried out on the possibility of using copolymers and cross-linking polymers for this purpose [35].

Due to such features as high resistance to bases and acids, good solubility in various organic solvents, thermal and physical stability, excellent film-forming ability and relatively low price, PVC is widely used in the production of various membranes [36]. It has been shown that PVC dissolved in an organic solvent (usually tetrahydrofuran) and used in combination with different carriers and plasticizers allows obtaining flexible, homogeneous membranes, which indicates its important advantage: good compatibility with other membrane components [35]. Cellulose triacetate (CTA, usually dissolved in dichloromethane), also commonly used in PIM formulation, has similar properties, although it is slightly less resistant to harsh environmental conditions than PVC [21,35]. Many PIMs recently applied for metal ion removal from aqueous solutions contain one of these polymers. Table 1 and Table 2 provide information on sample components (carriers and plasticizers/modifiers) of PIMs prepared during the last three years using PVC or CTA as base polymers and their applications for the transport/removal of different metal ions.

Some of the PIMs listed in Table 1 and Table 2 were used for the removal/transport of specific metal ions from model solutions, but they could also be potentially used for industrial-scale processes if the nature of industrial wastewater (e.g., pH, content of interfering components, etc.) corresponds to the optimal conditions/parameters of the efficient and selective laboratory-scale polymer inclusion membrane processes. After determining the optimal conditions for the laboratory membrane processes, many researchers have successfully used the designed PIMs to remove/recover metal ions from wastewater or leached waste [40,41,45,53,54,56]. Obtaining satisfactory effects of using PIMs for experiments performed on real wastewater significantly increases the probability that the membrane will also work on a larger industrial scale. However, in order to consider using a certain PIM in industry, it is necessary to check whether it is possible to repeatedly utilize the membrane with an acceptable performance. Despite the fact that in the case of many reports mentioned in Table 1 and Table 2 such studies have been carried out, and that it has been shown that designed membranes can be used several times [45,46,49,56], it is well known that the transport efficiency of PIMs fabricated using PVC and CTA decreases after repeated use. Such behavior is caused by the systematic leaching of the membrane components (carriers, plasticizers and modifiers) from the PIMs to the aqueous phases (i.e., feed and receiving phases) during their contact with the membrane, and it is related to inherent properties of these base polymers [35]. To increase the efficiency and stability of PVC- and CTA-based membranes and reduce the leaching of the membrane’s liquid phase during its reuse, various solutions are currently being used, including saturating the feed solution by the extractant used or doping the membranes with different nanoparticles, for example, reduced graphene oxide, ferrite Fe_3_O_4_, SiO_2_, TiO_2_ and silver nanoparticles [35,57,58,59]. However, not all such modifications bring the desired effect; in the case of transporting one component of the mixture, the use of a membrane with added nanoparticles may lead to an improvement in the efficiency of the process, while in the case of the other component a decrease in efficiency may be observed [58]. Another solution that significantly affects the properties of the PIM and the efficiency of the metal ion transport process is the application of a mixture of two polymers during membrane formulation. Recently, Tutkun and Kaparova [60] conducted selective separation of gallium ions from Zn, Co and Ni ions in the acidic solutions with a polymer inclusion membrane based on a CTA/PVC blend using trioctylphosphine oxide as the carrier and 2-nitrophenylpentyl ether as the plasticizer. They reported that although the proportions of all membrane components affect the efficiency of the process, the use of polymer blends as the base polymers could cause a positive synergy by increasing the membrane selectivity, extraction efficiency and the flux. The maximum extraction efficiency of gallium was 95.3%. The determination of optimal conditions for the membrane separation process based on utilization of a CTA/PVC PIM could be potentially relevant in the future for the recovery of gallium (a strategic metal used in the production of photovoltaic cells and computers) from waste/wastewater originating from zinc refineries and from bauxite alumina production [60].

Despite many advantages of PVC and CTA, they have limited use in specific environmental conditions (in highly alkaline conditions PVC dehydrochlorinates and CTA hydrolyses; CTA also hydrolyses in highly basic conditions) [35], which means that other polymers are sought to be used to formulate PIMs intended to work in harsh environments. One of the polymers characterized by high chemical and thermal stability as well as mechanical strength and excellent film-forming properties is polysulfone (PSF). Moreover, PSF exhibits economic feasibility [61], which is particularly important when considering the possibility of using PSF-based PIMs on an industrial scale. Recent results of experiments performed by Kunene et al. [62] regarding the use of a PSF-based PIM containing Aliquat 336 (tricaprylmethylammonium chloride, acting as both the carrier and the plasticizer) for the removal of chromium (VI) ions from model solutions with different pH (pH 2–12, adjusted by addition of NaOH solution) showed that the optimal composition membrane (40:60 *w*/*w*, PSF/Aliquat 336) with the highest extraction efficiency was chemically stable in both acidic and basic solutions and in temperatures as high as 180 °C. Unfortunately, despite the greater stability of PSF-based PIM, the Cr(VI) extraction efficiency was lower compared to the use of a traditional PVC-based PIM. Nevertheless, it is also important, especially for potential application of membranes containing polysulfone for the purpose of removing of chromium ions from wastewater, that PSF-based PIMs are more economical in terms of synthesizing (no plasticizer required).

In recent years, promising results have been obtained in the case of removal of various metal ions from acidic aqueous solutions with PIMs formulated using poly(vinylidene fluoride) (PVDF) as the base polymer. PVDF is characterized by high chemical resistance (resistance to acids, bases and strongly oxidizing substances), high mechanical strength and thermal stability. In addition, it exhibits high hydrophobicity compared to other polymers used in PIMs [35,63]. Sellami et al. [64] formulated a PIM based on PVDF, containing Aliquat 336 as the ion carrier and 2-NPOE as the plasticizer, and used it for transport of Cr(VI) ions from an acidic aqueous medium (HCl solution). Their results show that the PVDF-based PIM with 20 wt% of carrier allows for almost complete transport of Cr(VI) ions and that the addition of 5 wt% of plasticizer significantly increases the transport flux. What is also of great importance in relation to the potential application possibilities of the PVDF/Aliquat 336/2-NPOE membrane for the purpose of removing chromium ions from wastewater on an industrial scale is the fact that Cr(VI) ions were also selectively recovered with high efficiency (~97%) from a mixture containing other heavy metal ions (i.e., Cd(II), Pb(II), Fe(III), Zn(II), Cu(II), Ni(II) and Co(II)), and that the PIM showed high stability (above 190 h) and was suitable for long-term use. Huang et al. [65] applied a PVDF-based PIM with di(2-ethylhexyl) phosphinic acid (P227) as a carrier for the high-performance transport of lutetium (Lu(III)) ions from a model solution prepared by dissolving its oxide (>99.99%) in hydrochloric acid. They reported that the PIM showed a large difference in Lu(III) ion transport efficiency in the opposite transport direction and that the transport efficiency of lutetium ions increased with the duration of the membrane process (85% after 5 h and 96% after 12 h). A PVDF-based PIM with a P227 carrier has also been successfully used by Huang et al. [66] for selective Lu(III) removal from the hydrochloric acid solution (pH = 1.5) containing La(III) and Sm(III) ions. The recovery factor of Lu(III) ions was 91% after 36 h of membrane process, and additionally a process for regenerating PIMs was proposed and implemented. As the authors wrote: “it is expected that P227 @ PVDF PIMs have the potential to be applied to the grouped separation of rare earth elements (REE)”. The development of efficient methods based on the utilization of PIMs for the recovery of REEs is of great importance because of the increasing use of these metals in the modern industry (and the need to recover these metal ions from waste and wastewater) and because membrane processes are considered to be more environmentally friendly than other methods used for this purpose (e.g., precipitation, adsorption and solvent extraction) [67,68]. For many of the REEs (e.g., scandium), the currently dominant recovery methods from mining and metallurgical extractive industries as well as industrial wastes are various extraction methods, which usually involve the use of large amounts of toxic organic solvents [69]. A PVDF polymer has also been used by Liu et al. [70] in polymer inclusion membranes containing the new carrier, such as hydrophobic deep eutectic solvent (DES) for the recovery of Au(III) ions from a hydrochloric acid solution. Their results showed that the PVDF polymer has good compatibility with DES, and the formulated PIM had high reusability and selectivity and could achieve the quantitative extraction of Au(III) ions from mixed metal solutions. Extraction efficiency of gold ions was also impressive, as it exceeded 99% (92.4% after five consecutive transport experiments). Due to the excellent results of the application of new PVDF-DES PIMs, it can be assumed that similar membranes may be used on a larger scale in the future, e.g., for the recovery of gold from waste, e.g., waste electrical and electronic equipment.

PVDF is also frequently utilized in PIMs that contain copolymers as base polymers. The use of copolymers in membranes is aimed at improving their durability, stability and resistance to specific environmental conditions. Poly(vinylidene fluoride-co-hexafluoropropylene) (PVDF-HFP) is often used for this purpose. For example, Bahrami et al. [71] formulated PVDF-HFP based PIMs containing Cyphos IL 101 (trihexyltetradecylphosphonium chloride) or Aliquat 336 as metal ion carriers and used them for the removal of Cr(VI) from sulfate solutions (pH = 1.2). They found that both types of membranes can be used for chromium ion removal; however, a higher extraction efficiency of Cr(VI) by the Cyphos IL 101/PVDF-HFP PIM was observed in comparison with that achieved by the Aliquat 336/PVDF-HFP PIM. In addition, the mass loss during consecutive extraction/back-extraction cycles of the Cyphos IL 101 containing a PIM was significantly lower than that noticed in the case of PIMs with Aliquat 336. The application of Cyphos IL 101/PVDF-HFP PIMs also allowed for successful removal of Cr(VI) ions from chrome plating and well water, which indicates that the membrane may be potentially used on a larger scale in these industries in the future. Recently, Hedwig et al. [72], who applied polymer inclusion membranes consisting of PVDF-HFP as the base polymer, 2-NPOE as the plasticizer and DEHPA as the carrier for the selective scandium separation from real TiO_2_ production waste, reported high membrane efficiency (>60% of scandium was recovered with high selectivity and scandium mole fraction was more than two orders of magnitude higher in the receiving phase than in the original waste/feed phase) and concluded that the designed membrane could be an important tool for effective recovery of scandium from bulk waste. Gunasegaran et al. [73] used PVDF-Co-HFP based PIMs with different concentrations of Aliquat 336 as the membrane carrier for silver ion extraction from model aqueous solutions to assess what concentration of the carrier allows for obtaining the highest values of Ag(I) ion recovery. Determining the optimal conditions for the membrane process plays a key role in terms of the further use of this type of membrane, e.g., for the recovery of silver ions from real solutions/wastewater.

Obtaining a polymer inclusion membrane based on PVDF with improved mechanical and transport properties and increased efficiency, which extends its potential in the separation of heavy metals, may require much larger modifications of its composition. For example, Sellami et al. [74] formulated novel PVDF-based PIMs containing Aliquat 336 as the ion carrier and different types of montmorillonite clays (native sodium Cloisite Na^+^ (CNa)—silicate minerals characterized by low cost, high cation exchange capacity, swelling tendency, high aspect ratio and ease of chemical modification and organo-modified Cloisite 30B (C30B)) to study the influence of the nanoparticles’ nature and structure on the morphology of membranes and their usefulness for transport of toxic Cr(VI) ions. Transport experiments were performed for the feed phase containing chromium (VI) ions in 0.1 M HCl and the receiving phase buffered by a solution of acetic acid and ammonium acetate (pH = 5). Their results showed that the PIMs with 20 wt% of native sodium montmorillonite were stable during five cycles of the efficient Cr(VI) transport with limiting carrier loss due to the high membrane rigidity and hydrophobicity. Consequently, in the case of this type of PIM, incorporation of nanoparticles allows for extending the membrane lifetime (diminish the carrier loss) without losing its permselective properties.

Another solution to extend the lifetime/applicability of polymer inclusion membranes is the utilization of cross-linking polymers for their formulation. Hoque et al. [75] reported results of a first application of a cross-linked polymer inclusion membrane for enhanced gold recovery from aqua regia digests of electronic waste. In the research, they used membranes containing Cyphos IL 104 (trihexyltetradecylphosphonium bis (2,4,4-trimethylpentyl) phosphinate) as an ion carrier, CTA, PVC and PVDF-HFP were examined as base polymers, and additionally they checked the suitability of several chemical compounds as cross-linking agents (i.e., poly(ethylene glycol)dimethylacrylate (PEGDMA), poly(ethylene glycol)divinyl ether (PEGDVE) and N-ethylmaleimide (NEM)) and as initiators (i.e., triarylsulfonium hexafluorophosphate (TASHFP) and 2,2-dimethoxy-2-phenylacetophenone (DMPA)). Their results demonstrated that PIMs containing PVDF-HFP, PEGDMA and DMPA or TASHFP in strictly defined proportions allow for transporting of Au(III) ions from hydrochloric acid solutions twice as fast as corresponding non-cross-linked polymer-based PIMs, and that they are characterized by excellent stability over five successive transport experiments. In the case of aqua regia feed solutions, only a PIM based on cross-linking polymers with TASHFP was able to achieve complete Au(III) ion recovery (~100%). Because currently there is an intense search for fast and simple methods for recovering precious metals, including gold from WEEE waste, that are efficient, cost-effective and safe for the environment, and at the same time allow for conducting processes in highly acidic media [76], the polymer inclusion membrane processes described by Hoque et al. [75] as meeting most of these conditions may be used on a larger scale in the future. Similarly, it has been shown that the use of cross-linked polymer inclusion membranes formulated with the combination of most common base polymers (e.g., PVC, CTA and PVDF), several different photo-initiators and D2EHPA or Aliquat 336 as a carrier allowed for more efficient extraction of SCN^−^ and Zn^2+^ ions compared with that of corresponding non-cross-linked PIMs [77]. The results demonstrate the huge potential of cross-linking for enhancing the extraction performance of PIMs, as cross-linked membranes described in this study extracted up to 45% more of the corresponding target ions.

An interesting solution aimed at eliminating undesirable changes in membrane morphology during surface modification is the mixing of various polymers and polyelectrolytes in different proportions. For example, Bensaadi et al. [78] formulated CTA- and polycaprolactone (PCL)-based polymer inclusion membranes with D2EHPA as a specific carrier and with the addition of polyelectrolytes: polyvinylpyrrolidone (PVP), polyanetholsulfonic acid (PATSA), and polyethylene imine (PEI). In addition, 2-NPOE was added to the different membranes as the plasticizer. After examining their morphology, the membranes were used to transport chromium (VI) ions. It has been shown that the operating efficiency of the membranes differed significantly, as the chromium ion removal efficiency was 42% after 8 h of transport in the case of the CTA/PCL/PATSA/D2EHPA PIM and 43% for only 2 h using the PIM composed of CTA/PCL/PATSA/2-NPOE. However, the authors noted that the effectiveness of Cr(VI) ion removal did not exceed 50%, and assumed that the membrane deteriorated and lost its properties due to the hydrolysis of CTA under acidic conditions. Because the highest efficiency was obtained when the reception phase was ultrapure water, the designed membranes will not be suitable for wider industrial applications.

The presented examples of the utilization of various polymers for the purpose of formulating PIMs do not exhaust all possibilities in this regard. New polymers are systematically sought, the application of which can increase the efficiency of membranes with regard to the separation of specific metal ions. For example, Kazemzadeh et al. [79] used green polyol (synthesized by the reaction of CTA with epoxidized castor oil) as the base polymer to formulate a membrane containing 12-Crown-4 (12C4) as the carrier and 1-butyl-3-methylimidazolium chloride (BMIMCl), intended for the separation of lithium ions from an aqueous solution containing Li(I), Na(I), K(I), Ca(II) and Mg(II) ions. Including these metal ions in the conducted experiments is important because lithium is often found in brine reservoirs/wastewater in the company of alkali and alkaline earth metals, and its efficient recovery with the use of traditional methods, e.g., solvent extraction, is not easy. To improve the Li(I) ion enrichment process, Kazemzadeh et al. additionally employed the zeolitic imidazolate framework type ZIF-8, which increased the hydrophobicity and the roughness of the membrane. They reported that ZIF-8 nanoparticles have regular porosity and positive charge that can increase Li(I) ion flux and selectivity. The positive charge of ZIF-8 influences the divalent ion repulsion, and the average pore diameter of ZIF-8 particles that is larger than the lithium-ion diameter does not prevent the Li(I) ion transport across the membrane. Both phenomena have a strong impact on polymer inclusion membrane process selectivity. 

## 3. Polymer Inclusion Membrane Plasticizers

In most of the currently utilized polymer inclusion membranes plasticizers are used, the main role of which is to improve workability and flexibility of membrane structure. The plasticizers should be chemically stable substances, not leachable from the membranes during their operations, which means that they should not enter the feed/receiving phases. This feature is particularly important when PIMs are used to remove metal ions from industrial wastewater, which is often highly acidic or alkaline. As a consequence of the presence of the plasticizer, the obtained membrane should be characterized by appropriate softness and flexibility, and its operation should enable the effective transport of desired metal ions [25]. In the case of some PIMs, the role of the plasticizer may be played by ionic liquids (ILs), which are also used as ion carriers. This solution allows for limiting the amount of membrane components (membrane can only consist of base polymer and ILs) and it may have a positive effect on the wider scale industrial process costs, although the effect of ILs on PIM structure is not still fully understood [29]. On the other hand, it has been shown that even in the case of using ionic liquids with known and confirmed plasticizing properties (e.g., Aliquat 336), in PIM formulation the use of an additional plasticizer in a small amount (e.g., 2-NPOE) may contribute to a significant increase in metal ion transport efficiency [64].

One of the most frequently used plasticizers of polymer inclusion membranes in recent years is 2-NPOE, which is characterized by high dielectric constant and low viscosity [38,46,49]. It is known that these parameters influence the stability of complexes formed by metal ions in the membrane phase, but the performance of the plasticizer also depends on the properties of other components of the membrane. Often, 2-NPOE is used in PIMs containing PVC as the base polymer, because plasticizer molecules can easily bond with PVC chains and can provide a solvent-like environment for transporting the carrier–metal ion complexes in the membrane phase [80]. The plasticizer “neutralizes” the polar groups of a polymer with its own polar groups or contributes to increasing the distance between the polymer chains and reducing the influence of intermolecular forces [81]. The results of experiments performed by Hu et al. [82] based on the application of a PIM composed of PVC as the skeleton, 2-NPOE as the plasticizer and commercial LIX84I as the carrier for transport of Cu(II) ions showed not only a huge dependence of membrane composition on transport efficiency but also indicated that the significant membrane structure transition occurred while increasing the 2-NPOE content. The use of the small-angle X-ray scattering technique allowed for disclosing of microchannels with an average diameter of about 9 nm for a PIM containing 40 wt.% PVC, 30 wt.% 2-NOPE and 30 wt.% LIX84I, and such well-organized pathways in the membrane were essential for the efficient transport of Cu(II). It has been shown that 2-NPOE can also be used successfully in CTA-based PIMs. For example, Meziani at al. [83] used ionic liquids Aliquat 336 and tetradecyl(trihexyl)phosphonium chloride (THTDPCl) as carriers in PIMs containing CTA as the base polymer and 2-NPOE as the plasticizer, and applied the designed membranes for Bi(III) ion recovery from hydrochloric acid solutions and reported that both membranes enabled the quantitative recovery of bismuth ions. Additionally, the application of a CTA/Aliquat 336/2-NPOE PIM for Bi(III) transport from solutions containing a mixture of Bi-Mn-Sb-Pb ions led to efficient Bi(III) ion separation from Mn(II), while Sb(III) and Pb(II) were co-transported without affecting the effectivity of the PIM in terms of bismuth ion recovery. The development of separation methods intended for recovering technology-critical elements (TCEs) such as bismuth from different sources (e.g., industrial wastewater) is very significant. Recently, Szczepański et al. [84] examined the applicability of CTA/2-NPOE PIMs containing new reactive ionic liquids (RILs) based on the imidazole derivatives as a carrier for Cd(II) ion removal from multicomponent aqueous chloride solution also containing Cu(II), Pb(II) and Zn(II) ions. They found that from the three synthesized RILs only one of the longest alkyl chains can be used as the efficient carrier for Cd(II) ion removal and that the selectivity order independent of the experimental conditions was Cd(II) > Zn(II) > Pb(II) ≫ Cu (II). Kończyk and Ciesielski [85] applied CTA/2-NPOE PIMs with 1,8,15,22-tetra(1-heptyl)-calixresorcin[4]arene and its tetra- and octa-substituted derivatives containing phosphoryl, thiophosphoryl or ester groups as ion carriers for Pb(II) ion removal from aqueous nitrate solutions. They observed competitive transport of Pb(II) over Zn(II), Cd(II) and Cr(III) ions across PIMs under the optimal conditions and found that the Cr(III) ion presence in the feed phase disturbs effective re-extraction of Pb(II). Importantly, in Meziani’s et al. [83], Szczepański’s et al. [84] as well as Kończyk and Ciesielski’s [85] studies, the effectiveness of the developed membranes in the recovery of desired metal ions from polymetallic solutions was examined, which suggests that these PIMs may also be useful in the removal of metal ions from multicomponent industrial wastewater (this requires further research, however). Moreover, all these membranes were characterized by good stability. Compatibility of 2-NPOE with various carriers, including new ones (e.g., RILs) that have not been used so far for this purpose, may contribute to an even wider use of this plasticizer, also in membranes used on an industrial scale. Confirmation of this assumption may be the results reported by Zwierucha et al. [54], who used CTA/2-NPOE PIMs containing a calix[4]pyrrole derivative as an the ion carrier to dispose Hg(II) ions from industrial wastewater. The highest Hg(II) ion separation efficacy of 91.8% was obtained. The authors wrote that “optimally designed PIMs could be an interesting option for the industrial wastewater treatment due to the high removal efficiency of Hg(II) and great repeatability”. CTA/2-NPOE PIMs (with commercial carrier Cyanex 921) have also been successfully used for the removal of toxic As(V) ions from acid mine drainage (separation efficiency of 90%), which is globally recognized as one of the environmental pollutants of priority concern due to high concentrations of heavy metals and sulfates [56].

Another plasticizer frequently used in the last three years is 2-nitrophenyl pentyl ether (2-NPPE), characterized by lower viscosity and higher dielectric constant than 2-NPOE. It has been reported that the effectiveness of PIMs containing 2-NPPE may be slightly better in some cases, but due to its price, 2-nitrophenylpentyl ether is often replaced with 2-NPOE [25]. The results of many studies have shown that 2-NPPE allows for imparting appropriate properties to CTA-based membranes containing various carriers, such as 2-alkylimidazoles [50,53], acetylacetone derivatives [51,52] and calix[4]pyrrole and derivatives [55]. A significant part of CTA membranes formulated with the use of 2-NPPE have been successfully used to remove metal ions from model solutions with a composition corresponding to specific wastewater or directly from wastewater. For example, Radzymińska et al. [53] applied CTA/2-NPPE PIMs with 1-hexylimidazole (1) or 1-hexyl-2-methylimidazole (2) as carriers for the separation of silver(I) and zinc(II) ions from model nitrate solutions and from a solution obtained after leaching silver-oxide waste batteries. They reported that in the case of PIMs doped with (1), the recovery of both ions was high, whereas PIMs doped with (2) allowed for the recovery of Zn(II) ions that was almost two times higher than that of Ag(I) (both in the model and real solutions). Therefore, they proposed that the separation of Zn(II) and Ag(I) by the membrane technique should be carried out in two steps: in the first step, Zn(II) should be recovered using a PIM containing (2); in the second step, a PIM doped with (1) should be used for Ag(I) recovery. This solution may be potentially important in the future for the separation of silver and zinc ions from various types of waste (i.e., leachate of silver-oxide waste batteries). Nowik-Zając et al. [55] have shown that CTA/2-NPPE PIMs with a calixpyrrole ester derivative as the membrane carrier, due to their appropriate properties, might be a promising approach for selective extraction of Ag(I) ions from multi-metal ion solutions containing Cu(II), Pb(II), Cd(II), Ni(II), Zn(II) and Co(II) ions, such as copper smelting wastewater.

Bis(2-ethylhexyl)adipate (DEHA) is another commercially available plasticizer that is very slightly soluble in water and used mostly in polymer inclusion membranes based on PVC [86]. The results of several examinations showed that PVC-based PIMs with DEHA and various carriers (e.g., with acetylacetone, salen and Cyphos IL 101) are characterized by adequate plasticity and stability, and can be used for efficient removal and separation of different metal ions [40,42,43,45]. Recently, PVC/DEHA PIMs with the new extractant 2,6-bis(4-methoxybenzoyl)-diaminopyridine [87] have been applied for the recovery of noble metal ions, such as Au(III), Ag(I), Pd(II) and Pt(II) from model, highly acidic aqueous solutions. The obtained percentages of metal ions removal after 24 h of membrane processes were Ag(I)—94.89%, Au(III)—63.46%, Pt(II)—38.99% and Pd(II)—23.82%, whereas in the desorption processes (after 48 h) the highest percentages of recovery were observed for gold and silver ions (over 96%). Such a good performance of this novel PIM with 2,6-bis(4-methoxybenzoyl)-diaminopyridine in separation of silver and gold ions indicates its potential practical applicability for recovery of these noble metal ions from aqueous solutions. The possibility of using this membrane in a highly acidic environment, typical for solutions obtained as a result of leaching (by aqua regia) of WEEE waste, is of great importance. However, for a PVC/DEHA/2,6-bis(4-methoxybenzoyl)-diaminopyridine PIM to be used on a larger scale, e.g., in industry, further research is required regarding membrane stability, the possibility of its regeneration and effective working time. The recovery of valuable materials from WEEE, the amount of which is systematically growing, is currently one of the key problems for economic and environmental reasons (observed reduction in the amount of metal ores and the need to process waste), and new solutions are being sought in this field [88]. Polymer inclusion membranes containing bis(2-ethylhexyl)adipate as a plasticizer and other carriers have also been used for the recovery of noble metals ions, e.g., a PVC/DEHA /Cyphos IL 101 PIM was successfully used for the recovery of Ag(I), Pd(II) and Au(III) from leached (by concentrated nitric(V) acid and aqua regia) computer pins [45]. Moreover, since the regenerated membrane has proved to be effective in recovering noble metal ions several times, the chances of this solution for industrial applications are considerable.

Despite the wide use of various plasticizers in polymer inclusion membrane formulation, research is still being conducted to determine physical and chemical parameters affecting the performance of these components in membranes with a specific composition intended for the transport/separation of specific metal ions. Understanding these relationships is important for the further development of membrane techniques and their wider industrial applications. For example, Mancilla-Rico et al. [89] compared the effectiveness of membranes containing CTA as the support, Ionquest^®^ 801 ((2–ethylhexyl acid)-mono(2–ethylhexyl) phosphonic ester) as the extractant and two different plasticizers, 2-NPOE or TBEP (tri(2–butoxyethyl phosphate), to learn more about the relationship between membrane performance, nature and quantity of plasticizer. They noted that plasticizer characteristics such as low volatility, low viscosity, high dielectric constant, low cost and low toxicity are important. The last two characteristics are particularly relevant considering the possibility of using a plasticizer in membranes intended for operation on an industrial scale (economical and environmental reasons). After the examination of structure, electric resistance, dielectric constant, thickness, components’ distributions and stability, the obtained membranes were used to transport In(III) ions. Indium (III) is gaining more and more interest due to its applicability in electronic devices; however, it is associated with the necessity of metal recovery from electronic waste. The authors reported that although 2-NPOE presents less dispersion and affinity in the formulated PIMs and a plasticizer effect at higher content compared to TBEP, in the case of using a CTA/2-NPOE/Ionquest^®^ 801 PIM the increase in permittivity with In(III) ion sorption was more noticeable and higher permeability values were obtained. In(III) ion transport occurs more efficiently in well-plasticized membranes with a chemical environment of high polarity, although their stability is much lower due to minor affinity among the membrane components.

The obtained results are an excellent example, confirming that the appropriate selection of membrane components including plasticizer is not easy, and even in the case of poor compatibility of all components the membrane may effectively fulfil its role in the removal/recovery of specific metal ions. On the contrary, in the case of combinations of certain polymers and carriers, the addition of a plasticizer to the polymer inclusion membranes may not significantly improve their properties. For example, Kazemi and Yaftian [38] performed several experiments to examine the influence of various plasticizers, such as tri-n-butyl phosphate (TBP), tris(2-ethylhexyl)phosphate (TEHP), dibutyl phthalate (DBP) and 2-NPOE, on the stability and effectiveness of PIMs based on PVC and containing D2EHPA as the carrier, utilized for the transport of Bi(III) ions from sulfate solutions. They reported that the presence of the examined plasticizers in PIM composition did not influence the Bi(III) ion transport efficiency, revealing the absence of a synergetic effect. Interestingly, the results also showed that the plasticizer viscosity is relatively insignificant in improving the flux through the examined PIMs. Usually, however, analyzing the suitability of several plasticizers with regard to properties and effectiveness of PIMs allows for identifying one plasticizer, the use of which allows for obtaining a stable and flexible membrane that can be successfully used for the selective transport of specific metal ions from a specific solution [44,47]. Determining the optimal membrane composition (including the type and quantity of plasticizer) is crucial with regard to its feasibility for the recovery of metal ions on an industrial scale.

## 4. Carriers Used in PIMs

As the carriers used in polymer inclusion membranes play a key role in the processes of metal ion transport and separation, new compounds that can be used for this purpose are systematically sought. The research concerns both the possibilities of using in PIMs new chemical compounds, which so far have not been used in solvent extraction or in membrane processes, and the capabilities of utilization of well-known extractants as carriers in polymer inclusion membranes. In principle, chemicals that have proven to be successful as extractants in solvent extraction (SE) have the potential to act as carriers in PIMs as well, although their performance in membrane processes due to the influence of many more factors may not be identical to that in SE [24,43,87]. The proper choice of carrier has a significant impact on membrane effectiveness in relation to transport and separation of specific metal ions, but it is also important to properly design the membrane structure (selection of polymer matrix and plasticizer) in which the transported species can easily transfer on the fixed carrier [90].

The properties of carriers may be the basis for their division, with the most commonly used classification assigning them to one of the following groups: acidic and chelating (e.g., acidic: organophosphorus acids, such as D2EHPA, thiophosphorus acid esters, carboxylic acids and sulfonic acid compounds; chelating: Lix 841, acetylacetone and its derivatives), basic (e.g., quaternary ammonium salt such as Aliquat 336, pyridine and its derivatives and alkylimidazoles), macrocyclic and macromolecular (e.g., calixarenes, crown ethers, calix crowns and cyclodextrine) and neutral and solvating (e.g., Cyphos 101 and 104) [25]. The choice of the appropriate carrier depends on the characteristics of the analyte to be transported, e.g., if anions are to be removed, suitable extractants are, for example, those that are able to form ion pairs [24]. Carriers from all of these groups have been used to remove various metal ions, and many research results have shown that they can potentially be utilized to remove these ions from multi-component model solutions and real wastewater. This section presents examples of the use in the last three years of well-known, commercially available carriers and new chemical compounds acting as carriers in PIMs intended for the removal of metal ions, with particular emphasis on those that can be potentially used in industry.

One of the most frequently used carriers in recent years is di-(2-ethylhexyl)phosphoric acid (D2EHPA), which belongs to the group of acidic carriers. The application of D2EHPA in polymer inclusion membranes for metal ion transport involves exchange of metal ions with hydrogen ions from carrier molecules [25]. D2EHPA’s great advantage is its compatibility with various membrane components. For example, this carrier has been used in PIMs formulated with the use of different polymers as the membrane matrix (i.e., PVC [38,39], CTA [48,49], cross-linked polymers (combination of PVC, CTA and PVDF) [77] or mixtures of various polymers and polyelectrolytes [78]) as well as with or without plasticizers (i.e., with 2-NPOE [49] or without plasticizers [38,39]), and the obtained membranes were sufficiently stable and flexible to be applied in PIM processes. PIM experiments described earlier have been performed for various metal ions: Bi(III) [38,39], Cu(II) [48], Zn(II) [77] and Cr(III) [78]. Additionally, the newly synthesized PVC/D2EHPA PIM containing silver nanoparticles has been used to extract trace metal ions (Co(II), Ni(II), Cu(II) and Cd(II)) in polluted water and proved to be a promising tool for transport of the target metals; however, the performance improvement is required with respect to stability of both the carrier and nanoparticles in the membrane [59]. Recently, Hammache et al. [91] examined the possibility of using D2EHPA in membrane processes aimed at the recovery of neodymium (Nd) and praseodymium (Pr), contained in the Nd-Fe-B magnets of end-of-life computer hard disk drives (HDDs). Both Nd(III) and Pr(III) belong to rare earth elements, whose recovery from WEEE is extremely important due to their numerous applications in the high-tech sector. Researchers examined the performance of four polymer membranes prepared by blending CTA and PEI (polyethylenimine) with the addition of one of the following compounds, D2EHPA, TDDA (tridodecylamine), TOA (trioctylamine) or TOPO (trioctylphosphine oxide); in addition, diluted HDD leachates were used as feed solutions. Results of diffusion dialysis experiments conducted with the use of these four membranes have shown that the transfer of Nd(III) and Pr(III) cations was very small through all membranes, whereas boron, which was present in feed solutions in the form of neutral boric acid molecules, was transferred to a greater extent, especially through membranes with high water uptake (with TDDA or TOA). A clean and energy-efficient process, such as diffusion dialysis, has great potential for the recovery of neodymium and praseodymium from WEEE and may be potentially used in the future on a larger industrial scale. However, the use of membrane processes for REE recovery from WEEE also has some limitations, e.g., poor separation and selectivity, energy consumption and the remaining large amount of acidic or alkaline wastewater (result of leaching) [67]. Therefore, further research is needed both with regard to the possibility of using various membranes with different compositions as well as to the optimization of REE recovery processes. Further research is also needed to minimize the impact of the developed methods on the environment and the risk to living organisms [92].

It has been demonstrated that the function of efficient carriers in PIMs intended for the removal and selective transport of specific metal ions can be performed by chelating compounds, such as acetylacetone and its derivatives. These compounds were used, among others, in the processes of removing the following metal ions from various solutions: Zn(II), Cu(II), Cr(III), Ni(II) (acetylacetone, [40]), Zn(II), Ni(II), Cr(III)(3-propyl-pentane-2,4-dione, [41]), Zn(II), Cr(III) and Ni(II) (ethylenodiamino-bis-acetylacetone, [51]). Moreover, Radzymińska-Lenarcik and Witt [40] compared the performance of acetylacetone in three different processes: liquid–liquid solvent extraction, transport through polymer inclusion membranes containing PVC and di(2-ethylhexyl)adipate as a plasticizer and measurement of sorption on polymeric sorbents (based on PVC). They reported that all applied methods of metal ion separation were comparably effective. However, in the case of using acac-doped PIMs, the extraction coefficients of Zn(II), Cu(II) and Cr(III) ions were as high as 94%, 78% and 50%, respectively, whereas Ni(II) ions were scarcely transported across membranes (9%). The authors also showed that these techniques are efficient in removing heavy metals from small amounts of contaminated soil leached with ammonia. The applicability of a derivative of acetylacetone as a carrier in PIMs intended for removing metal ions from real wastewater samples was also demonstrated. For example, 3-propyl-pentane-2,4-dione used as carrier in PIMs allowed for efficient removal of metal ions from three different types of post-galvanic wastewater, containing zinc or nickel or nickel–chromium ions. However, it was shown that the recovery of metal ions was even higher when this compound was used in the sorption process [41]. Because acetylacetone and its derivatives have proven to be effective metal ion carriers, their properties are well known, and their synthesis is relatively cheap and simple, it can be assumed that in the future there will be research on the possibility of their use in PIMs for recovery of various metal ions on a larger scale, e.g., from industrial wastewater.

The group of organic compounds containing heteroatoms that have free electronic pairs and can easily form complexes with various metal ions includes N,N’-bis(salicylidene)ethylenediamine (salen, a polydentate ligand with oxygen and nitrogen donor atoms) and its derivatives. Witt and coworkers [42] examined salen as an extractant (solvent extraction) or as a carrier (polymer inclusion membranes) for the recovery of Ni(II), Cu(II) or Zn(II) ions from single-component, model aqueous solutions, and reported that this compound is a very effective extractant, especially for removing copper(II) ions, but its efficiency depends on its concentration in the system. They also noted that PVC/DEHA /salen membranes can be successfully used as sorbents for the recovery of subject metal ions from solutions, but transport of these metal ions across membranes was not efficient. The results of the use of salen for the recovery of noble metal ions (Pd(II), Ag(I), Pt(II) and Au(III)) from model single-component and multi-component aqueous solutions by solvent extraction and membrane processes have shown that this compound is an effective extractant as well as a carrier. Extraction efficiency was over 99% for all noble metal ions in single-component solutions and over 94% in multi-component solutions, whereas the percentage of the sorption of metal ions (PIMs) from single-component solutions was the highest for silver ions (93.23%) and for multi-metal solutions for Pd(II) ions (92.96%). Additionally, it has been reported that salen can form various types of metal ion complexes (different L:M ratios) in the examined solutions [43]. Due to the high values of the recovery of noble metal ions from multi-component solutions with the use of PVC/DEHA/salen PIMs, it can be assumed that after further research on the stability/regeneration of such membranes, this method may be used on a larger industrial scale. The ability of salen derivatives to bind gold ions was also confirmed by the results obtained by Campo-Cobo et al. [93], who synthesized salen-type ligands with electron-accepting substituents on the aromatic ring and applied them as carriers in polymer inclusion membranes intended for the extraction of various metal ions (i.e., Cu(II), Pb(II), Ca(II), Al(III), Co(II), Au(III), Fe(II), Fe(III), Ni(II), Mn(II) and Zn(II)) from aqueous solutions. They found that all PIMs containing salen derivatives had higher selectivity to gold than to other metals and allowed for the extraction of acceptable percentages of this metal, with the percentage of extracted gold depending on the type of substituent present in the ligand’s aromatic ring, the pH of the feed phase and the area of the membrane. The obtained polymer inclusion membranes were used for up to three cycles of gold extraction before they degraded. However, the evidence of metal ion transport through formulated PIMs was not found. The results of both studies [43,93] indicate that, regardless of the mechanism of the process, salen is particularly effective as a component of PIMs for gold, palladium and silver ion recovery, and the effective recovery of these ions from WEEE leachate is very important. An advantage of salen and its derivatives in terms of their potential use on an industrial scale is also the relatively easy and inexpensive process of their synthesis and characterization [93].

Compounds with confirmed metal ion extraction abilities that have recently been used in polymer inclusion membranes as basic carriers are alkyl derivatives of imidazoles. Depending on their structure, alkylimidazoles may behave differently in relation to various metal ions. It has been shown that their properties can be modified by changing the size of the alkyl groups and their positions in the imidazole ring, and what is important in relation to the wide application possibilities of these compounds is that their synthesis is relatively simple and inexpensive. As a result, metal ion carriers with desired properties (e.g., hydrophobic) can be easily “designed”. Until now, these compounds have been successfully used primarily as carriers in PIMs for the transport and separation of nonferrous metal ions, such as Zn(II), Cu(II), Cd(II), Co(II) and Ni(II) [50,94]. Importantly, the research on the possibility of utilization of alkylimidazoles was conducted both with the use of model solutions and feed phases obtained as a result of the leaching of waste. For example, the separation of Ag(I) and Zn(II) ions was examined with the use of PIMs containing 1-hexylimidazoles or 1-hexyl-2-methylimidazoles as ion carriers in solutions obtained as a result of a spent battery with silver–zinc cell leaching [53]. Because even relatively small differences in the structure of these compounds (e.g., the presence of an additional methyl group attached to the imidazole ring [53]) may significantly affect their suitability as carriers in PIMs intended for the removal of specific metal ions and because the membrane process is also influenced by many other factors (e.g., content of other substances in the feed phase, pH, membrane composition, etc.), it is necessary to precisely determine the optimal process conditions for a specific type of carrier/wastewater before specified alkylimidazole will be used on an industrial scale.

The pyridine derivatives, which can form various complexes with different metal ions [95,96], and many of which were used in other methods for metal ion removal [97,98], were also applied during the last three years as carriers in PIMs. As already mentioned, 2,6-bis(4-methoxybenzoyl)-diaminopyridine has been successfully used for the recovery of noble metal ions [87]. Recently, Qiu et al. [44] examined the usefulness of four modified 2-aminomethylpyridine derivatives with hydrophobic alkyl chains, including 2-[N-(tert-butyloxycarbonylmethyl)-2-picolyamino]acetate, N,N-dioctyl-2-aminomethylpyridine, tert-butyl 2-(N-octyl-2-picolyamino)acetate and N,N-didecyl-2-aminomethylpyridine as carriers in PVC-based polymer inclusion membranes containing various plasticizers (DOP, DEHA or NPOE), applied for Cu(II) ion recovery. They reported that the hydrophobic modification of 2-aminomethylpyridine derivatives can boost the selective transport of copper(II) ions through PIMs and increase membrane stability. As revealed by small-angle X-ray scattering analysis, the generation of microchannels in PIMs has been induced by such modification, and consequently contributed to the enhanced Cu(II) flux. The other three pyridine derivatives, 2,6-bis((benzoyl-R)amino)pyridine, where R = H, 4-Me or 4-NMe_2_, were used as carriers in PVC/DEHA PIMs intended for the recovery of copper(II), nickel(II), cobalt(II) and zinc(II) ions from model aqueous solutions [99]. It was shown that these compounds, whose synthesis was relatively simple and cheap, and whose structure confirmation (NMR and HRMS) did not raise any doubts, exhibit high complex-forming properties and can play the role of carriers. However, as noted by the authors, in order to use these pyridine derivatives for the removal of metal ions from wastewater, further research on the increase in efficiency of membrane processes and membrane stability (e.g., use of other plasticizers/polymers) is needed.

Many studies were based on the use of well-known, commercially available basic carriers, such as Aliquat 336, for removing various metal ions, and based on ensuring that the optimization of the conditions of performed membrane processes were efficient and selective. An important advantage of this carrier, confirmed by many research results, is its compatibility with various polymer inclusion membrane components, both polymers (e.g., PSF [62], CTA [83], PVDF [64], PVDF-HFP [71,73] and cross-linked polymers PVC/CTA/PVDF [77]) and plasticizers (e.g., 2-NPOE [64]). It should be emphasized that Aliquat 336 contained in PIMs can also successfully perform both functions of a carrier and a plasticizer [62]. PIMs containing Aliquat 336 as a carrier were used, for example, to remove Cr(VI) [62,64,71], Ag(I) [73], Zn(II) [77] and Bi(III) [83] ions from various solutions. Fontas et al. [100] analyzed the influence of Aliquat 336 and its two derivatives AlqNO_3_ and AlqSCN and a type of polymer (PVC or CTA) on membranes morphology and changes in PIM morphology due to contact with water. Accurate characteristics (morphological and chemical) of the membrane surface are important, as they significantly affect the entire process. In addition, they investigated the effect of membrane composition on arsenic(V) ion transport. They reported that as a result of contact with water, a partial loss of the carriers has been observed (largest in the case of a PIM AlqCl—CTA) and that the nature of carrier has a great influence on the morphology of the membranes (AlqCl was more miscible with the polymer matrix, whereas other carriers formed nano-spheres on the surface of the PIMs). It has been shown that Aliquat 336 is also suitable for removing V(V) ions from spent alumina hydrodesulfurization catalysts, suggesting its future applicability on a larger scale for real waste/wastewater treatment [37]. Currently, studies are also carried out to optimize the composition of PIMs containing Aliquat 336 in terms of their various environmental applications related to the transport of As(V) ions with the use of response surface methodology (RSM) coupled to the Derringer’s desirability function (DF) [101].

The macrocyclic and macromolecular carriers category includes, among others, compounds such as calixarenes, crown ethers and calix crowns. The growing interest in these carriers results from their high separation selectivity and ability to form stable complexes with various species, both cations and anions as well as neutral molecules [25]. Zawierucha et al. [54], who used calix[4]pyrrole derivatives as carriers in PIMs, indicated another significant advantage of these compounds, namely well-developed, relatively easy methods of their synthesis and the possibility of simple modifications in their structure (introduction of appropriate substituents), which may favorably influence their complexing properties. calix[4]pyrrole derivatives were utilized in polymer inclusion membranes both to remove metal ions from model solutions (e.g., Ag(I) ions from nitrate aqueous solutions [55]) and from industrial wastewater (e.g., Hg(II) ions, [54]). PIMs obtained using these compounds were highly permeable, but the membrane processes depended on many factors (i.e., amount of carrier, composition of feed and receiving phases, etc.), and in order to obtain high recovery values of the metal ions, it was first necessary to establish the optimal process conditions. Recently, it has also been shown that calixresorcin[4]arene derivatives can be utilized as efficient carriers in PIMs. Zawierucha et al. [102] used such compounds to transport Cr(VI) ions from acidic media and reported that under optimized conditions, the chromium ion removal efficiency was as high as 98.4%. Konczyk and Ciesielski [85], who examined performance of various calixresorcin[4]arene derivatives as carriers in polymer inclusion membranes intended to the transport of Pb(II) ions, stated that the obtained results create a new perspective for further work on PIMs with these metals ion carriers. However, they also wrote that improving the efficiency of metal ion extraction and the membrane stability is of key importance in the perspective of applicability of PIMs with calixresorcin[4]arene derivatives on a larger scale, e.g., in water and wastewater treatment plants.

Cyphos IL 101, 102 and 104 (ionic liquids) are some of the well-known and frequently used chemical compounds belonging to the group of neutral and solvating carriers [25,103]. During the last three years, Cyphos IL 101 and 104 have been applied among others as carriers in different PIMs intended for the recovery of gold ions from WEEE [45,75]. Makowka and Pospiech [104] successfully used Cyphos IL 104 as a carrier in CTA/2-NPOE PIMs for separation of Ce(III) ions from a solution containing La(III), Cu(II), Co(II) and Ni(II) ions. Both noble metals and rare earth elements are valuable raw materials important in many industries, and WEEE is considered as one of their key secondary sources. Hence, intensive research is conducted to develop efficient, environmentally friendly methods, in which membrane techniques, including PIMs, play an important role [105]. The advantage of Cyphos IL 101, 102 and 104 ionic liquids is, in addition to being efficient as PIM carriers, easy availability (commercially available) and compatibility with various membrane components (polymers and plasticizers) [25,45,75].

In addition to using new chemical compounds as carriers or optimizing the conditions for conducting membrane processes with the utilization of known extractants, in order to increase the selectivity and efficiency of PIMs, two different carriers can be used simultaneously. For example, Zeng et al. [106] used novel polymer inclusion membranes based on CTA, containing 2-ethylhexyl phosphonic acid mono 2-ethylhexyl (P507) and tributyl phosphate (TBP) as the carriers for lithium and magnesium ion separation from salt-lake brines with high Mg(II)/Li(I) ion ratios. They reported that CTA/P507-TBP PIMs retained excellent selectivity (in seven consecutive transport experiments) and permeability, proving a potential for long-term operation, and a strategy of integrating P507-TBP carriers could help develop sustainable and highly stable PIMs for extracting lithium from natural salt-lake brines. Xu et al. [107] applied a mixture of 1-butyl-3-methylimidazolium bis(trifluoromethylsulfonyl)imide ([C4mim][NTf2]) and tributylphosphate (TBP) as the carrier in a CTA-based PIM for separation of Li(I) and Mg(II) ions. They noted that the formulated PIM displayed high stability in the conducted membrane extraction process and that TBP-[C4mim][NTf2] was an efficient carrier for Li(I) ions. These methods may have wider application in the future, as the separation of lithium and magnesium ions is crucial for the exploitation of lithium resources with a high concentration of magnesium ions. Paredes and de San Miguel [108] reported that CTA-based PIMs with LIX-54-100 and Cyanex 923 as carriers and without the addition of a plasticizer were able to selectively extract and concentrate Li(I) ions from diluted alkaline aqueous media as well as from natural seawater.

**Figure 3:**
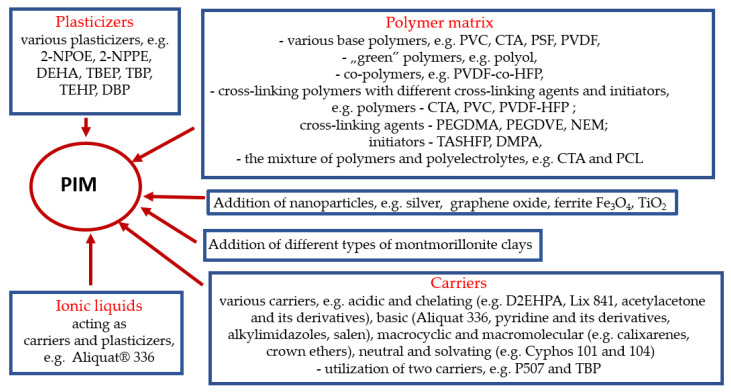
The main modifications made over the past three years during the formulation of PIMs [prepared on the basis of the references contained in this article].

Figure 3 shows a summary of the main modifications made over the past three years during the formulation of PIMs.

## 5. Conclusions

In recent years, polymer inclusion membranes have been intensively used for the removal of various metal ions from model aqueous solutions, both single-metallic and multi-metallic. The achievements related to the use of PIMs result primarily from the possibility of using various chemical compounds for their formulation, and this applies to all polymers constituting the membrane “skeleton”, carriers responsible for binding metal ions and plasticizers giving membranes appropriate flexibility. The observed tendency in relation to the polymer matrix is the use of not only single, well-known polymers previously utilized in PIMs (e.g., PVC and CTA), but also the application of novel copolymers and cross-linking polymers, which in turn may lead to the enhancement of membrane stability. In the case of carriers, attempts are made to use well-known extractants, previously utilized in solvent extraction (e.g., acetylacetone), but more and more often new chemical compounds are “designed”, synthesized and utilized as carriers in PIMs intended for specific metal ion removal (e.g., derivatives of alkylimidazoles, pyridine, Aliquat 336, calixresorcin[4]arene, etc.). The group of compounds used as plasticizers is also systematically expanding, but the possibility of reducing the amount of plasticizers is also being investigated (especially when ionic liquids are used in PIM formulation), which may have a positive economic impact on membrane processes. Another promising solution for increasing the performance of membranes is the addition of other substances, such as reduced graphene oxide or silver nanoparticles. However, designing of a polymer inclusion membrane to remove particular metal ions from specific solutions with different properties (e.g., various pH and presence of other substances) is a multivariate approach. The first stage of the research concerns the proper selection of the membrane components (the possibility of using various polymers, plasticizers, carriers and “additives”), and after the membrane formulation, it is necessary to check its properties, including its strength and stability (e.g., whether it is homogeneous, whether it is flexible, whether there is leaching of membrane components under specific experimental conditions or whether the membrane can be used multiple times over a longer period of time). The use of a formulated membrane should allow for efficient and selective transport of the desired metal ions from the feed phase to receiving phase, which is usually relatively easily achieved with simple model solutions containing two different metal ions. Multi-metal solutions or solutions containing other interfering substances may require more complex procedures, e.g., a two-step membrane processes based on the use of two different carriers. An important limitation of the use of PIMs is also the possibility of fouling of the membranes and the related necessity to frequently replace them or develop an effective method of their regeneration (which extends the process time and increases its costs). Although the polymer inclusion membrane processes themselves are not long or labor-intensive and can be carried out in mild and harsh conditions, due to the diversity of the factors that influence them, determining the optimal process conditions is not easy.

The examples included in this work clearly demonstrate that PIMs with a properly selected composition and under optimal conditions of the membrane processes are versatile and have the potential to be used on an industrial scale. In many cases, the described PIMs have also been successfully used to remove toxic metal ions from real wastewater or to recover precious metal ions from leached WEEE, which confirms the huge capability of PIMs for industrial applications. An important advantage of PIMs is also the use of small amounts of toxic solvents and not generating large amounts of sludge, which makes the technique eco-friendly, especially with comparison to traditionally used methods (e.g., solvent extraction and adsorption). However, further research is necessary to solve the problem of membrane stability (e.g., inhibition of carrier leaching) and the possibility of their multiple use (development of cheap and efficient methods of membrane regeneration). Another consideration that may be of importance when using PIMs on a larger scale is the development of environmentally friendly and cost-effective methods for handling used membranes.

## Figures and Tables

**Table 1:** Examples of PIMs prepared using PVC as base polymer and their applications for removal/transport of various metal ions.

Carrier/Plasticizer	Type of Metal Ions/ Feed Solution	Main Advantages	Year of Publication/ Reference
Aliquat^®^ 336/ oleic acid	V(V)/ model solution containing vanadium ions and Na_2_SO_4_ (pH = 2.5) and model solution with V(V) and the mixture of Al(III), Co(II), Cu(II), Fe(II), Mn(II) and Ni(II) ions (composition corresponding to leachate of spent alumina hydrodesulfurization catalysts).	PIM enables the selective and efficient extraction of V(V) ions from both single- and multi-metal ion solutions (extraction of ~73% and ~71% (in two-step process) of V(V) ions, respectively). Potentially suitable for removing V(V) ions from spent alumina hydrodesulfurization catalysts.	2022 [37]
D2EHPA/ TBP, TEHP, DBP, and 2-NPOE	Bi(III)/ model solutions containing Bi(III) ions and Na_2_SO_4_ (pH = 1.3) and model solutions with Bi(III) and Cd(II), Co(II), Cu(II), Mn(II), Ni(II), Zn(II), Al(III), Cr(III), Fe(III) and Mo(VI) ions.	The presence of TBP, TEHP, DBP or 2-NPOE in the PIM composition did not affect the transport efficiency. PIM (with no plasticizers) demonstrated selectivity towards Bi(III) over Cd(II), Co (II), Cu(II), Mn(II), Ni(II), Zn(II), Al(III), Cr(III), Fe(III) and Mo(VI). PIM was stable in 10 transport experiments.	2022 [38]
D2EHPA	Bi(III)/ model sulfate solution containing Bi(III) ions (pH = 1.3) and model Bi(III) solution with Cu(II), Zn(II), Mn(II), Co(II), Ni(II), Cd(II), Al(III), Cr(III) and Fe(III) ions with addition of NaF.	A plasticizer-free PIM extracted with high selectivity Bi(III) ions from single- and multi-metal ion solutions (extraction of ~99% and from ~98% to ~61% (depending on the composition of the mixture) of bismuth ions, respectively). PIM exhibited excellent performance stability in 15 extraction/back-extraction cycles.	2021 [39]
Acetylacetone/ DEHA	Zn(II), Cu(II), Cr(III) and Ni(II)/ model solutions (pH = 7.8) containing ammonia and metal amounts corresponding to those determined in samples originating from the roadside soil belts.	The designed PIM allowed for effective removal of all examined metal ions from the solution, except for nickel ions. The extraction coefficients of Zn(II), Cu(II), Cr(III) and Ni(II) were 94%, 78%, 50% and 9%, respectively. PIM can potentially be used for removal of heavy metals from the soils located along expressways.	2020 [40]
3-propyl-pentane-2,4-dione/ DEHA	Zn(II), Ni(II) and Cr(III)/ three different types of post-galvanic wastewater containing zinc or nickel or nickel–chromium ions (pH = 7.28, 7.32 and 7.97).	PIM allowed for the effective removal of zinc and chromium ions (75–78% of Zn(II) and 62–64% of Cr(III) ions); however, proper treatment of wastewater required frequent replacement of PIMs.	2020 [41]
Salen/ DEHA	Ni(II), Cu(II) and Zn(II)/ model basic aqueous solutions containing metal ions and ammonia (pH = 12.5).	Sorption on polymer membranes with salen as a carrier was especially effective for removing copper(II) ions from aqueous solutions (extraction of Cu(II) ions from ~99% to ~67%, depending on experimental conditions). PIMs were not suitable for the transport of the investigated metal ions.	2020 [42]
Salen/ DEHA	Pd(II), Ag(I), Pt(II) and Au(III)/ model single- and multi-metal solutions containing metal ions and ammonia (pH = 12.5).	The effectiveness of polymer membranes with salen was very high for sorption processes. The percentages of sorption were 93.23% for Ag(I) ions, ~75% for Au(III) ions, ~69% and ~66% for Pd(II) and Pt(II) ions in single-component solutions ~93%, ~84%, ~81% and ~48% for Pd(II), Au(III), Ag(I) and Pt(II) ions in multi-metal solutions. Desorption processes were also effective, and membranes were used several times.	2021 [43]
2-amino-methylpyridine derivatives/ DOP, DEHA or 2-NPOE	Cu(II)/ model aqueous solutions with metal ions and hydrochloric acid (pH = 4.5).	The hydrophobic modification of 2-aminomethylpyridine derivatives boosted the selective transport of copper ions by designed PIMs and improved the stability of membrane.	2021 [44]
Cyphos IL 101/ DEHA	Ni(II), Zn(II), Co(II), Cu(II), Sn(II), Pb(II), Ag(I), Pd(II) and Au(III)/ solution obtained by leaching computer pins with concentrated nitric (V) acid and aqua regia.	Polymer films allowed for the efficient recovery of precious metals (~99% of Au(III), ~79% of Ag(I) and ~64% of Pd(II)) and after regeneration can be successfully used several times.	2021 [45]

Where Aliquat^®^ 336—trioctylmethylammonium chloride, TBP—tri-n-butyl phosphate, TEHP—tris(2-ethylhexyl) phosphate, DBP—dibutyl phthalate, D2EHPA—bis(2-ethylhexyl)phosphoric acid, 2-NPOE—2-nitrophenyl n-octyl ether, DOP—dibutyl phthalate, DEHA—bis(2-ethylhexyl)adipate, salen—N,N’-bis(salicylidene)ethylenediamine, Cyphos IL 101—trihexyl(tetradecyl)phosphonium chloride.

**Table 2:** Examples of PIMs prepared using CTA as base polymer and their applications for removal/transport of various metal ions.

Carrier/Plasticizer	Type of Metal Ions/ Feed Solution	Main Advantages	Year of publication/ Reference
SF/ 2-NPOE	Ni(II), Cd(II), Co(II), Pb(II), Cu(II) and Mg(II)/ model solutions with metal ions and nitric acid (pH = 5).	Metal cations were effectively transported through the PIM in the order of Ni(II) > Ca(II) > Co(II) > Pb(II) > Cu(II) > Mg(II). Excellent durability of PIM (5 days of successive metal cation transportation).	2020 [46]
TOA/ TBP, 2-NPOE, DBP or TEHP	Bi(III)/ model solutions with various ions, including metal ions and hydrochloric acid.	High selectivity of PIMs towards Bi(III) ions (extraction efficiency > 97%) over Cu(II), Pb(II), Zn(II), Ni(II), Co(II), Cd(II), Fe(III), Cr(III), Mo(VI), W(VI), NO_3_^−^ and SO_4_^2−^.	2022 [47]
D2EHPA/ acetylated kraft lignin as filler, no plasticizer	Cu(II)/ model solutions with metal ions and nitric acid (pH = 4.5).	High stability of PIM and efficient recovery of Cu(II) (transport of 74% of copper ions)	2020 [48]
D2EHPA/ 2-NPOE	Cr(III)/ model solution containing different interfering ions (SO_4_^2−^, Cl^−^, Na^+^, K^+^ and Ca^2+^) and HCl (pH = 4).	Efficient recovery of Cr(III) ions (~96%), slightly lower in the presence of interfering ions, potentially a future application of the developed system to real samples.	2022 [49]
2-alkylimidazoles (alkyl = methyl, ethyl, propyl and butyl)/ 2-NPPE	Cu(II), Zn(II), Cd(II) and Ni(II)/ model solutions with the mixture of metal ions and tetra-methylammonium hydroxide (pH = 6).	Efficient recovery of metal ions decreasing in sequence Cu(II) > Zn(II) > Co(II) > Ni(II). Recovery of Cu(II) ions from ~87% to ~99%, depending on the type of 2-alkylimidazole.	2021 [50]
Ethylenodiamino-bis-acetylacetone/ 2-NPPE	Zn(II), Cr(III) and Ni(II)/ model solutions with the mixture of metals ion and tetramethylammonium hydroxide (pH = 7.8).	Effective separation of Zn(II) ions from aqueous solutions of Zn(II), Cr(III) and Ni(II) ions. The recovery factors of Zn(II), Cr(III) and Ni(II) were 90%, 65% and 6%, respectively.	2020 [51]
Ethylenediamine-bis-acetylacetone (EDAB-acac)/ 2-NPPE	Zn(II), Co(II), Ni(II) Cu(II) and Cd(II)/ model solutions with the mixture of metal ions and ammonia buffer (pH = 7.8).	Separation of Zn(II) from a mixture of nonferrous metal ions. The transport selectivities of the PIMs were Zn(II) > Cd(II) > Co(II) > Cu(II) > Ni(II). The recovery factors for Zn(II) ions were from 90% to 98%, depending on experimental conditions.	2020 [52]
1-hexylimidazole and 1-hexyl-2-methylimidazole/ 2-NPPE	Zn(II) and Ag(I)/ model solutions with the mixture of metal ions and tetra-methylammonium hydroxide (pH = 6.5), solutions after the leaching of a spent battery with a silver–zinc cell	Separation of Ag(I) and Zn(II) ions in the transport process from both model and real solutions. Two-step process enabling the efficient recovery of both metal ions. The recovery coefficients (leaching solution) of Ag(I) and Zn(II) for PIMs doped with 1-hexylimidazole were 86% and 90%, whereas for PIMs with 1-hexyl-2-methylimidazole were 47% and 94%, respectively.	2020 [53]
Meso-octamethyl-calix[4]pyrrole/ 2-NPOE	Hg(II)/ model solutions with the metal ions and hydrochloric acid and wastewater from the zinc smelter	PIM was stable, reusable and showed a good performance in selectively removing of mercury ions. The highest Hg(II) ion separation efficiency was ~92% for model solution and ~86% for zinc smelting wastewater.	2022 [54]
Calix[4]pyrrole/ 2-NPPE	Ag(I)/ model acidic solutions containing metal ions (pH = 4.0)	PIMs might be a promising approach for selective extraction of Ag(I) (extraction efficiency ~92%) from a mixed solution of Cu(II), Pb(II), Cd(II), Ni(II), Zn(II) and Co(II), e.g., from copper smelting wastewater.	2020 [55]
Cyanex 921/ 2-NPOE	As(V)/ synthetic aqueous leachates (sulfuric acid solutions) and real acid mine drainage (AMD)	Good performance of the PIM in the selective removal of arsenic, both from model solution (extraction efficiency ~96%) and AMD (extraction efficiency 90%). The membrane can be used repeatedly to separate arsenic ions.	2020 [56]

Where SF—surfactin, TOA—trioctylamine, TBP—tri-n-butyl phosphate, TEHP—tris(2-ethylhexyl) phosphate, DBP—dibutyl phthalate, D2EHPA—bis(2-ethylhexyl)phosphoric acid, 2-NPOE—2-nitrophenyl n-octyl ether, 2-NPPE—o-nitrophenyl pentyl ether, Cyanex 921—tri octyl phosphine oxides.

## Data Availability

Not applicable.
